# Supplementary material for: Direct real-time RT-PCR for the detection of dengue virus from patient serum in Lao PDR
Source: PLoS One. 2025 Aug 18;20(8):e0330459. doi: 10.1371/journal.pone.0330459 (PMC12360527; doi:10.1371/journal.pone.0330459)
Supplement: S1 Table — (DOCX) [file pone.0330459.s001.docx]

Table S1. Results of Luna RT-qPCR performed on neat and 1/10 diluted DENV2 isolate solutions.

|  | | **Direct Luna RT-qPCR (Cq)** | | | |
| --- | --- | --- | --- | --- | --- |
| **Template** | **Not diluted** | | **1/10 dilution** | |  |
| DENV2 10^-1^ | 19.01 | 19.65 | 23.77 | 24.28 |  |
| DENV2 10^-2^ | 22.12 | 21.79 | 26.94 | 26.48 |  |
| DENV2 10^-3^ | 25.78 | 25.50 | 29.35 | 30.28 |  |
| DENV2 10^-4^ | 28.95 | 29.91 | 33.16 | 33.62 |  |
| DENV2 10^-5^ | 32.32 | 32.50 | 36.34 | 37.01 |  |
| DENV2 10^-6^ | 37.27 | 35.01 | neg | 38.16 |  |
| DENV2 10^-7^ | neg | neg | neg | neg |  |
| DENV2 10^-8^ | neg | neg | neg | neg |  |
| Pos (high) | 21.44 | 21.45 | 21.64 | 21.42 |  |
| Pos (medium) | 24.98 | 24.78 | 24.48 | 24.86 |  |
| Pos (low) | 27.95 | 27.80 | 27.91 | 28.22 |  |

Luna RT-qPCR was performed directly (without prior RNA purification) on DENV2 isolate dilutions (10^-1^ to 10^-8^). Neg = negative result, no Cq; Pos= synthetic RNA in three concentrations (high, medium and low) used as RT-qPCR positive control. Liquid primers (400nM) and liquid probe (200nM) were used for Luna RT-qPCR.
