# Supplementary material for: Direct real-time RT-PCR for the detection of dengue virus from patient serum in Lao PDR
Source: PLoS One. 2025 Aug 18;20(8):e0330459. doi: 10.1371/journal.pone.0330459 (PMC12360527; doi:10.1371/journal.pone.0330459)
Supplement: S2 Table — (DOCX) [file pone.0330459.s002.docx]

Table S2. RT-qPCR results (Cq values) for direct Luna RT-qPCR and gold standard RT-qPCR process (with prior RNA purification) from patient sera.

| **DENV1**  **N=29** | | | **DENV2**  **N=27** | | | **DENV3**  **N=22** | | | **DENV4**  **N=24** | | | **NEGATIVE**  **N=30** | | |
| --- | --- | --- | --- | --- | --- | --- | --- | --- | --- | --- | --- | --- | --- | --- |
| **SS III** | **Luna** | | **SS III** | **Luna** | | **SS III** | **Luna** | | **SS III** | **Luna** | | **SS III** | **Luna** | |
| **RNA** | **Diluted serum** | **Non-diluted serum** | **RNA** | **Diluted serum** | **Non-diluted serum** | **RNA** | **Diluted serum** | **Non-diluted serum** | **RNA** | **Diluted serum** | **Non-diluted serum** | **RNA** | **Diluted serum** | **Non-diluted serum** |
| 19.22 | 22.20 | 19.75 | 19.24 | 20.09 | 17.76 | 26.32 | 24.81 | 35.04 | 21.61 | 22.10 | 18.92 | No Cq | No Cq | No Cq |
| 20.19 | 22.84 | 21.17 | 19.36 | 20.50 | 18.07 | 27.11 | 28.57 | No Cq | 22.86 | 24.33 | 22.40 | No Cq | No Cq | No Cq |
| 20.94 | 23.05 | 21.63 | 21.32 | 21.19 | 19.31 | 29.27 | 28.89 | No Cq | 23.4 | 24.6 | 22.97 | No Cq | No Cq | No Cq |
| 21.81 | 23.47 | 22.05 | 22.30 | 23.09 | 21.79 | 31.02 | 29.11 | No Cq | 23.64 | 24.67 | 23.20 | No Cq | No Cq | No Cq |
| 23.21 | 24.29 | 23.22 | 24.09 | 23.11 | 26.98 | 31.63 | 30.69 | No Cq | 24.28 | 25.16 | 23.37 | No Cq | No Cq | No Cq |
| 23.68 | 24.89 | 23.23 | 26.05 | 25.46 | 27.64 | 31.88 | 31.33 | No Cq | 24.62 | 25.19 | 26.97 | No Cq | No Cq | No Cq |
| 24.27 | 26.04 | 26.45 | 26.24 | 25.83 | 29.19 | 32.11 | 31.75 | No Cq | 24.77 | 25.29 | 42.88 | No Cq | No Cq | No Cq |
| 24.62 | 26.27 | 26.53 | 26.36 | 26.87 | 29.48 | 32.20 | 32.12 | No Cq | 26.02 | 26.81 | No Cq | No Cq | No Cq | No Cq |
| 24.92 | 27.25 | 28.40 | 27.67 | 27.47 | 33.19 | 33.66 | 33.38 | No Cq | 26.52 | 26.88 | No Cq | No Cq | No Cq | No Cq |
| 25.50 | 27.41 | 28.74 | 28.03 | 28.05 | 35.15 | 34.16 | 33.90 | No Cq | 27.05 | 27.40 | No Cq | No Cq | No Cq | No Cq |
| 26.03 | 29.22 | 30.14 | 28.63 | 28.54 | No Cq | 35.26 | 34.86 | No Cq | 27.22 | 27.52 | No Cq | No Cq | No Cq | No Cq |
| 27.06 | 29.46 | 30.46 | 28.65 | 29.24 | No Cq | 36.29 | 35.09 | No Cq | 27.77 | 28.10 | No Cq | No Cq | No Cq | No Cq |
| 27.43 | 30.06 | 30.63 | 29.21 | 29.27 | No Cq | 37.05 | 35.13 | No Cq | 28.46 | 28.79 | No Cq | No Cq | No Cq | No Cq |
| 28.08 | 31.08 | 34.36 | 32.48 | 31.15 | No Cq | 37.46 | 35.95 | No Cq | 30.07 | 29.37 | No Cq | No Cq | No Cq | No Cq |
| 31.01 | 31.67 | 35.05 | 33.45 | 34.05 | No Cq | 37.50 | No Cq | No Cq | 32.53 | 32.48 | No Cq | No Cq | No Cq | No Cq |
| 31.23 | 33.54 | No Cq | 34.79 | 35.00 | No Cq | 39.15 | No Cq | No Cq | 32.60 | 32.51 | No Cq | No Cq | No Cq | No Cq |
| 31.49 | 33.86 | No Cq | 36 | 36.25 | No Cq | 39.52 | No Cq | No Cq | 34.35 | 34.60 | No Cq | No Cq | No Cq | No Cq |
| 32.58 | 33.86 | No Cq | 36.04 | 36.46 | No Cq | 40.25 | No Cq | No Cq | 34.53 | 34.83 | No Cq | No Cq | No Cq | No Cq |
| 33.61 | 34.76 | No Cq | 36.37 | 39.46 | No Cq | 40.55 | No Cq | No Cq | 35.02 | 35.27 | No Cq | No Cq | No Cq | No Cq |
| 33.94 | 34.89 | No Cq | 36.71 | 40.68 | No Cq | 40.68 | No Cq | No Cq | 35.05 | 35.57 | No Cq | No Cq | No Cq | No Cq |
| 34.04 | 35.10 | No Cq | 37.07 | 40.75 | No Cq | 41.19 | No Cq | No Cq | 36.37 | 37.77 | No Cq | No Cq | No Cq | No Cq |
| 34.44 | 35.34 | No Cq | 38.31 | No Cq | No Cq | 41.55 | No Cq | No Cq | 37.22 | 40.01 | No Cq | No Cq | No Cq | No Cq |
| 35.48 | 35.34 | No Cq | 38.74 | No Cq | No Cq |  |  |  | 37.23 | 40.61 | No Cq | No Cq | No Cq | No Cq |
| 35.93 | 35.46 | No Cq | 39.42 | No Cq | No Cq |  |  |  | 39.76 | No Cq | No Cq | No Cq | No Cq | No Cq |
| 36.93 | 37.94 | No Cq | 39.47 | No Cq | No Cq |  |  |  |  |  |  | No Cq | No Cq | No Cq |
| 37.81 | 39.63 | No Cq | 39.95 | No Cq | No Cq |  |  |  |  |  |  | No Cq | No Cq | No Cq |
| No Cq | No Cq | No Cq | No Cq | No Cq | No Cq |  |  |  |  |  |  | No Cq | No Cq | No Cq |
| No Cq | No Cq | No Cq |  |  |  |  |  |  |  |  |  | No Cq | No Cq | No Cq |
| No Cq | No Cq | No Cq |  |  |  |  |  |  |  |  |  | No Cq | No Cq | No Cq |
|  |  |  |  |  |  |  |  |  |  |  |  | No Cq | No Cq | No Cq |
| Positive control | | | Positive control | | | Positive control | | | Positive control | | | Positive control | | |
| 23.51 | 22.62 | 21.91 | 23.51 | 22.62 | 21.91 | 22.55 | 23.12 | 23.64 | 23.51 | 22.62 | 21.91 | 22.55 | 23.12 | 23.64 |
| 27.17 | 25.59 | 24.59 | 27.17 | 25.59 | 24.59 | 26.07 | 26.07 | 26.87 | 27.17 | 25.59 | 24.59 | 26.07 | 26.07 | 26.87 |
| 30.45 | 29.09 | 29.01 | 30.45 | 29.09 | 29.01 | 31.22 | 29.21 | 29.80 | 30.45 | 29.09 | 29.01 | 31.22 | 29.21 | 29.80 |

In the table are displayed Cq value obtained. SS III= reference standard process including RNA purification followed by SuperScript III RT-qPCR. Luna= direct Luna RT-qPCR. PCR templates were 132 patient sera, non-diluted or diluted 1/10 for Luna RT-qPCR. DENV1= sera that were found positive for dengue serotype 1 by RT-qPCR at the time of diagnostic; DENV2= sera that were found positive for dengue serotype 2 by RT-qPCR at the time of diagnostic; DENV3= sera that were found positive for dengue serotype 3 by RT-qPCR at the time of diagnostic. DENV4= sera that were found positive for dengue serotype 4 by RT-qPCR at the time of diagnostic. NEGATIVE= sera that were found negative (No Cq) by DENV RT-qPCR at the time of diagnostic. Luna=Luna RT-qPCR kit. Grey shade corresponds to negative results (No Cq or Cq>40).
