# Supplementary material for: Direct real-time RT-PCR for the detection of dengue virus from patient serum in Lao PDR
Source: PLoS One. 2025 Aug 18;20(8):e0330459. doi: 10.1371/journal.pone.0330459 (PMC12360527; doi:10.1371/journal.pone.0330459)
Supplement: S3 Table — (DOCX) [file pone.0330459.s003.docx]

Table S3. Sensitivity of direct Luna RT-qPCR from 1/10 diluted sera for the detection of DENV in comparison to reference standard process (RNA purification followed by SSIII RT-PCR) according to the DENV serotype.

|  |  | Sample found positive by reference standard process (RNA purification followed by SSIII RT-qPCR) | | | |
| --- | --- | --- | --- | --- | --- |
|  |  | DENV1 | DENV2 | DENV3 | DENV4 |
| Direct Luna RT-qPCR | Positive | 26 | 21 | 14 | 21 |
|  | Negative | 0 | 7 | 3 | 3 |
|  | Total | 26 | 28 | 17 | 24 |
| Sensitivity (95%CI) | | 100% (86.8-100) | 75% (55.1-89.3) | 82.3% (56.6-96.2) | 87.5% (67.6-97.3) |

DENV1= sera that were found positive for dengue serotype 1 by RT-qPCR at the time of diagnostic; DENV2= sera that were found positive for dengue serotype 2 by RT-qPCR at the time of diagnostic; DENV3= sera that were found positive for dengue serotype 3 by RT-qPCR at the time of diagnostic. DENV4= sera that were found positive for dengue serotype 4 by RT-qPCR at the time of diagnostic. Negative = No Cq or Cq>40.
